# Evaluation of Sex Differences in Preclinical Pharmacology Research: How Far Is Left to Go?

**DOI:** 10.3390/ph16060786

**Published:** 2023-05-24

**Authors:** Sarah Allegra, Francesco Chiara, Daniela Di Grazia, Marco Gaspari, Silvia De Francia

**Affiliations:** Department of Biological and Clinical Sciences, University of Turin, S. Luigi Gonzaga Hospital, 10043 Orbassano, Italy; 336124@edu.unito.it (F.C.); daniela.digrazia@unito.it (D.D.G.); marco.gaspari@unito.it (M.G.); silvia.defrancia@unito.it (S.D.F.)

**Keywords:** preclinical experimentation, pharmacology, sex differences

## Abstract

Until the last quarter of the 20th century, sex was not recognized as a variable in health research, nor was it believed to be a factor that could affect health and illness. Researchers preferred studying male models for a variety of reasons, such as simplicity, lower costs, hormone confounding effects, and fear of liability from perinatal exposure in case of pregnancy. Equitable representation is imperative for determining the safety, effectiveness, and tolerance of therapeutic agents for all consumers. Decades of female models’ underrepresentation in preclinical studies has resulted in inequality in the understanding, diagnosis, and treatment of disease between the sexes. Sex bias has been highlighted as one of the contributing factors to the poor translation and replicability of preclinical research. There have been multiple calls for action, and the inclusion of sex as a biological variable is increasingly supported. However, although there has been substantial progress in the efforts to include more female models in preclinical studies, disparities today remain. In the present review, we consider the current standard practice of the preclinical research setting, why the sex bias exists, why there is the need to include female models, and what risks may arise from continuing this exclusion from experimental design.

## 1. Therapeutic Agent Development Is a Significant Challenge

Therapeutic agent development is driven by medical need, disease prevalence, and the likelihood of success. Therapeutic agent candidate selection is an iterative process between chemistry and biology, whose aim is to refine the molecular properties until a compound suitable for advancing to humans is found. It is an expensive, long, and high-risk process which takes from 10 to 15 years, and it is associated with a high attrition rate; 80 to 90% of research projects fail before they ever get tested in humans, and for every therapeutic agent that gains Food and Drug Administration (FDA) approval, more than 1000 were developed but failed. Prior to administration to humans, the pharmacology and biochemistry of the therapeutic agent is established using an extensive range of in vitro and in vivo test procedures during the preclinical studies [1]. Indeed, starting from the study of the medicine effects on cell cultures, the process goes through animal experimentation, ending with its use in the human species in different clinical phases. It is also an FDA regulatory requirement that the therapeutic agent is administered to animals to assess its safety. Later-stage animal testing is also required to assess the carcinogenicity and the effects on the reproductive system. The goal of preclinical studies is to accurately model, in animals, the desired biological effect of a medicine. This allows doctors to predict the treatment outcome in patients, and to identify toxicities associated with a therapeutic agent, with the aim of predicting adverse events in people [2].

There has been a revolution within clinical trials to include females in the research pipeline. However, there has been limited change in the preclinical arena, yet the preclinical research lays the groundwork for the subsequent clinical trials. In 1993, the Council for International Organizations of Medical Sciences proposed a law, approved by the FDA, requiring women to be included in clinical trials funded by the National Institutes of Health (NIH). Women of “child-bearing potential” were indeed excluded from clinical trials until 1993; until 1993, zero women have ever been enrolled in clinical trials. The NIH issued a mandate in 2014 to guarantee that both male and female subjects are represented in preclinical studies [3]. A comparable program by the Canadian Institute of Health that mandated inquiries about sex and gender during the application process for research funding resulted in a marked rise in the number of applications that took into account both the male and female sexes (from 26 to 48%) [4]. They made a point to mention that scientists working in the biological field were the least likely to admit to including sex in their study. The prospects for policy measures to address sex bias are highlighted by these findings. Unfortunately, not all research is sponsored through these channels, and the inclusion of both the male and female sexes in research pipelines is not yet achieved.

Kim and colleagues looked at the sex annotation of cells in pertinent articles published in the same journal in 2018 to determine how far the reporting of cell sex in studies has come since the examination in 2013. A total of 53 of the 107 papers describing cell experiments reported the sex of the cells; 12 studies only used female cells, 18 used both male and female cells, and 23 only used male cells. Cell lines were employed more frequently than primary cells, which resulted in more instances of sex omission. More than half of the investigations in the articles discussing trials utilizing mouse primary cells solely employed male cells [5]. In other studies, the authors described the 10-year results and lessons discovered from applicant forms, the development of resources for applicants and evaluators, and the grant review requirements in an effort to inform the implementation of scientific policy. The group included all participants in 15 Canadian Institutes of Health Research contests that were started by investigators between 2011 and 2019, as well as grant evaluators between 2018 and 2019. A total of 39,390 applications have been submitted since 2011. The percentage of reports integrating gender and sex increased from 12 to 33% and 22 to 83%, respectively. Applications in population health research gave gender the most consideration (82%). Applications with main investigators who were female were more likely to integrate sex (and gender) than those with male principal investigators throughout every competition. Since 2018, applications with strong sex integration scores and high gender integration scores have a higher chance of receiving funding. Qualitative observations showed that sex and gender were frequently confused [6]. A series of publications look at the preclinical research setting in existing normative practice, the drive to include women, and the reasons behind the discrepancy. They investigate organizational change theory as a tool for developing the institutional and individual strategies required to alter the present situation to establish a scientific environment where sex-sensitive methods are automatically implemented in preclinical research [7,8,9].

## 2. Methods of Research

The search was performed in PubMed (https://pubmed.ncbi.nlm.nih.gov/, accessed on 1 November 2022) databases starting from November 2022. The following terms were used to find papers that were pertinent: “sex”, “preclinical studies”, “pharmacokinetics”, “pharmacodinamics”, “animal models”, “cellular culture”, “male”, and “female” and “transegender”. The considered timeline is 1973-–2022. Through article search, approximately 1600 papers were initially identified; duplicate and unrelated articles were deleted. Of the 89 remaining articles after assessing the full text for eligible articles, 46 were excluded, leaving 43 in the study.

We looked through the references in the review articles to find any additional qualifying original manuscripts. Only English-language articles were chosen. In this review, we summarize the studies on sex evaluation in preclinical research pharmacological studies, sex differences in animal models used in pharmacological research, sex differences in cellular models used in pharmacological research, related adverse reactions, and patients’ age.

Clinical trial-related publications were eliminated since they failed to relate to the goal of the study that is presented.

## 3. Gender-Specific Pharmacokinetics and Pharmacodynamics

Women and men respond differently to treatments; this mainly depends on physiological, anatomical, and hormonal characteristics. The existence of the differences in therapeutic agent pharmacokinetics and pharmacodynamics influences the response to treatments [10,11,12,13,14,15,16,17,18,19,20,21,22,23,24,25,26,27,28,29,30,31,32,33,34,35,36,37,38,39,40,41,42,43,44,45]. Although this was already known since 1932, the year in which the first study on the gender difference in the pharmacology of barbiturates in rats was reported, full awareness of the relevance of the role of gender pharmacology only came at the end of the last century [16,26,27,46,47]. By pharmacokinetics, we mean the study of the following four phases of a medicine transition in the body: absorption, distribution, metabolism, and elimination. These four stages are primarily influenced by age and hormones, thus showing significant differences related to sex [48]. Sex hormones may interact negatively with drugs and their metabolic pathways through different mechanisms; these include absorption, competition for transporters, competition and/or regulation of expression of drug-metabolizing enzymes and sex steroids, and drug interactions with pharmacodynamics [49]. In females, drug effectiveness and adverse drug reactions may be impacted by variations in endogenous sex steroid hormones that happen naturally during the menstrual cycle, during pregnancy, and during the transition to menopause [50]. Exogenous hormones are additionally used by women as a contraceptive, as a treatment for hot flashes, nocturnal sweats, vaginal dryness, and a number of other conditions. Hence, these hormone therapies could be viewed as both medical treatments and a cause of adverse drug reactions, as well as a modulator of the effects of other medications. In other words, while drug metabolic pathways may have an impact on exogenous hormones used for therapy, exogenous hormones may also have an impact on other drugs via modifying metabolism. There may be variations in how each person reacts to exogenous hormones due to inter-individual variations in metabolic pathway components (pharmacogenetics) [49].

Male reproductive aging does not result in a complete cessation of testosterone production or spermatogenesis, unlike female reproductive aging (menopause) or organic androgen lack in males (caused by diseases of the brain, pituitary, or testes). In fact, the decrease in circulating testosterone levels brought on by aging in and of itself is moderate, and testosterone levels are typically in the low–normal range in men. However, a small percentage of aging men may experience testosterone deficiency, which is influenced by the presence of comorbidities. According to recent research, elderly men who maintain their health and fitness typically keep their serum testosterone levels regular. The terms andropause, viropause, partial androgen deficit in the aging male, and late-onset hypogonadism have all been used to describe age-related low testosterone in men [51]. Additionally, the gonadal axis is suppressed by the use of some medications, including glucocorticoids and opioids [52,53,54]. Considering pharmacogenetics, it has been observed that cells that carry the UGT1A4*1a allele may have decreased clearance of testosterone as compared to those with the *3a allele [55].

Pharmacodynamics, on the other hand, indicates the effect of a therapeutic agent on bodies and studies the biochemical and physiological effects and their mechanism of action. There are numerous pharmacodynamic differences depending on sex, mainly mediated by hormones, genes, and the environment. The primary organ for excreting drug metabolites or parent drug molecules is the kidney. All three of the main renal processes—glomerular filtration, tubular secretion, and tubular reabsorption—show documented sex differences. Men often have a higher renal clearance than women. The hepatic enzyme activity is altered by elevated levels of estrogen and progesterone, which can lead to an increased drug buildup or decreased drug clearance in some cases. Autoimmunity is influenced by prolactin and female steroid hormones [37]. The incidence and severity of autoimmune/inflammatory illnesses are two to ten times higher in females than in males due to the hypothalamic–pituitary–adrenal and hypothalamic–pituitary–gonadal axes’ regulation of immunity. Females of reproductive age are the ones who typically have autoimmune illnesses. Hormone levels that fluctuate during menstruation, when using oral contraceptives, during pregnancy, or throughout menopause can also affect metabolic alterations. For instance, some asthmatic women experience symptoms that intensify before or during their periods. Intensive physical activity has been linked to an increase in oxidative stress. Oxidative stress has been linked to gender disparities, particularly as people get older. Studies to investigate this have produced contradictory results, despite the fact that sex hormones are thought to have a major role in modifying sex-based differences in pharmacokinetics. Midazolam clearance, for example, which measures the CYP3A4 metabolic activity, did not change during the course of the menstrual cycle [56]; in addition, studies on eletriptan (used to treat migraines) also showed no changes in response to sex or menstrual cycle [57].

While the pharmacokinetic differences are simpler to analyze, the pharmacodynamic differences are more difficult to detect [26]. However, both deserve a worthy study in the preclinical phase in terms of gender differences, or the resulting clinical phase will be limited and approximate.

## 4. The Current Standard Practice of Pharmacological Preclinical Research

The main goal of the preclinical studies is to define the therapeutic agent pharmacokinetics and pharmacodynamics, anticipating the active level in the target compartment, to predict the safe starting dose and dose escalation scheme for phase 1 clinical trials. Preclinical research exhibits an endemic persistent sex bias that mostly concentrates on male animals [58,59]; this bias is still visible even when the disease of interest is a disorder that is more common in women. Yoon et al. discovered that only 12% of papers looking at illnesses common in women included studies of females or both the male and female sexes [60].

However, the gender bias is not only observed in the in vivo studies; the in vitro studies have historically ignored the relevance of sex of origin, and it is often still ignored. In the preclinical experimentation phase, the use of female animals is relatively low and the cells are usually considered asexual, since the sex of the donor is not reported, not considering, instead, that even cultured cells have a sex, at least during the first maintenance steps [58,61,62,63]. Despite this, pharmacodynamic differences are increasingly emerging, concerning increasingly relevant pharmacological targets [18,27,46,63,64,65,66]. Hormones have a key role in the modulation of pharmacodynamic reactions [49,67,68]; they regulate many functions within our organism, thanks to the interaction with specific intracellular receptors [69]. Estrogens and androgens behave differently, inducing highly variable responses from a molecular point of view [70,71,72,73]. Furthermore, hormonal changes depend on the age and reproductive life of the woman as they are conditioned by fertile age, pregnancy, postnatal period, and menopause [74]. In addition to hormones, genetics also regulate responses to treatment differently between the sexes.

However, recently, something has been changing. In 2018, Cvitanovíc Tomas and colleagues modified the SteatoNet in silico model to build the computer model LiverSex [75,76], which accounts for sex differences in the liver. Sex-related effects on growth hormone release are included in the data from estrogen and androgen receptor responses. The model has not yet been validated in people, but only in mice. In 2020, Thiele et al. developed sex-specific whole-body metabolic models [77]; 20 organs, 6 sex organs, 6 different blood cell types, systemic blood circulation, the blood–brain barrier, and the gastrointestinal lumen, including the microbiome, were used to illustrate the male and female physiologies.

## 5. Why the Sex Bias Exists (and It Is Very Robust)

In humans, sex is determined by sex chromosomes. The X and Y chromosomes carry different numbers and sets of genes; about 1000 genes are carried on the X chromosome, and only a few dozen genes are carried on the Y chromosome. A series of recombination events, followed by the loss of genetic material on the Y gene, leads to the morphological differentiation of the sex chromosomes [3]. Most genes on the sex chromosomes are not directly involved in sex determination, and the development of male or female, for a long time, has been mainly attributed to the presence of a single locus, the sex-determining region gene on the Y chromosome (SRY). Other studies have shown that this concept is much more variable than hypothesized, and alternative mechanisms can play a role on sexual development, widely different from that expected by the karyotype. Thus, there is growing proof that the sex-limited chromosome in some systems evolved independently and has no connection to the X or Z chromosomes. For instance, B chromosomes, which are small, unimportant chromosomes that frequently transmit selfishly, serve as the Y chromosome in *Rhinocola aceris* and *Cacopsylla peregrina* as well as a W chromosome in several Lake Malawi cichlids [78,79]. Strong evidence suggests that the *Lepidoptera W* chromosome evolved after the Z chromosome, likely from a B chromosome [80]. In the case of the pill bug (*Armadillium vulgare*), a Wolbachia feminizer that has been integrated into the nuclear genome gave rise to the W chromosome [81]. This opened the interesting potential that cytoplasmic male sterility factors, which are widespread in both insects and plants, may offer opportunities for the creation of non-homologous W chromosomes when they are transported to the nuclear genome.

Furthermore, the deep genetic difference between the sexes is also indicated by older studies. In 1961, Mary Lyon suggested that one of the two X chromosomes in females becomes genetically silent early in a female embryo’s development. Additionally, this happens randomly from cell to cell, connoting biological females with incredible genetic mosaicism [82].

A striking number of exceptions, and thus, a parallel diversity of underlying mechanisms, are revealed by the diversity of the sex chromosomes. This variability shows that the laws governing sex chromosomal evolution are complex and not universal. The systems that diverge or turn over most frequently may be the most instructive going forward, as comparisons can be made to separate cause from effect [83].

The International Mouse Phenotyping Consortium revealed that sex was a substantial source of variance within the control data and as a modulator of a treatment effect by analyzing data from 10 institutes, 14,000 wild type mice, and 40 thousand knockout mice for 234 features [84]. Sexually dimorphic effects in a wide range of biological systems highlights the importance that we should always examine both the male and female sexes and take sex into account as a source of variation.

Enzymes involved in therapeutic agent metabolism have sexually dimorphic expression patterns in a variety of species, which has an impact on its metabolism. In humans, the mRNA and protein measurements of the cytochrome p450 (CYP) 3A4 in the liver reveal higher levels in females [85]. Additionally, studies found that this enzyme is more active in females [86]. Rat and mouse livers exhibit a high level of sexually dimorphic gene expression [87,88,89,90,91]. For instance, rats express a male-specific CYP2C11 pattern, whereas the CYP2C12 is exclusive to women [92]. When comparing age-matched male and female mouse liver microsomes, the CYP1A2 was consistently more prevalent in males. Male and female mice 3 to 4 weeks of age had higher levels of hepatic expression of the CYP2B9 than mice of other ages, and the CYP2B9 was the only enzyme that was found in pregnant mouse liver microsomes at higher levels than age-matched females. It is interesting to note that only the kidney showed a sexually dimorphic expression of the CYP2B9, 2D26, 2E1, and 4B1 [93]. The complexity of the metabolic pathways emphasizes the significance of comprehending medicine exposure in each sex, at the correct time point, and in the relevant tissue when conducting pharmacodynamic assessments, even though rodent sex differences may not always transfer into similar patterns in humans.

The female estrous cycle is partly responsible for the exclusion of females from some research. Rats go through their estrous cycle for 4–5 days. Progesterone rises quickly, starting early in the metestrum phase of the post-ovulatory cycle on day 1, and falls quickly in the diestrum phase on day 2. Ovulation brings a significant increase in progesterone release and estrogen levels that occur during proestrum. When estrous occurs on day 4, the hormone levels return to normal after a transient temporary rise in estradiol [94]. It is believed that the estrous cycle introduces unpredictability into experimental procedures. The therapeutic agent responsiveness in females can also be impacted by hormonal fluctuations during the menstrual cycle [95,96]. To eliminate the effects of the estrous cycle, it was suggested that pharmacological tests be administered only during diestrum, that only male models be used, or that a counterbalanced design be used to average any changes [97]. Ovariectomy and castration procedures may be used to remove gonadal influences from the female and male rats to examine the effect of hormonal changes on experimental outcomes. To research the physiological effects of circulating hormones, these animals’ behavioral and neurochemical profiles can be compared to those of properly cycling females and intact males. Exogenous hormonal therapies, such as estradiol, progesterone, or both, and, if required, testosterone, may also be given to castrated and ovariectomized animals.

## 6. Excursus on Preclinical Pharmacology Research: Sex Disaggregated Data or Not?

Drug use and abuse differ in men and women [98,99,100]. Women seem to be more susceptible to drug use acquisition, maintenance, regulation, and relapse phases. This increased susceptibility may be facilitated by the ovarian cycle, as higher estrogen levels were linked to higher levels of illicit drug use among women [101]. The influence of sex and gonadal hormones on the behavioral and neurochemical reactions to addictive substances was better understood using animal models. In Table 1, we summarize the preclinical pharmacological studies which include sex evaluation.

Psychedelics, commonly referred to as hallucinogens, have an impact on sensory processing, perception, and cognition, primarily through the serotonin 5-HT2A receptor (5-HT2AR). As it relates to mental illnesses including depression and substance use disorders, this class of psychoactive drugs—which includes lysergic acid diethylamide, psilocybin, mescaline, and the substituted amphetamine 1-(2,5-dimethoxy-4-iodophenyl)-2-aminopropane (DOI)—is gaining increasing attention. Sex is frequently left out of research that examines the possible clinical effects of hallucinogenic drugs on human subjects. Rodent models have contributed much to our understanding of the pharmacology of psychedelics, yet most preclinical research has just looked at male mice. Jaster and colleagues examined the impact of DOI on male and female mice’s head-twitch behavior, a rodent behavioral model of the potential of psychedelic drugs in humans. In the C57BL/6J mice, DOI causes more ovariectomized behavior in females than in males, a sex-specific increased behavior that was not even seen in 129S6/SvEv animals. In both the male and female C57BL/6J mice, volinanserin, a 5-HT2AR antagonist, completely inhibited the DOI-induced ovariectomized behavior. There was no sex-related difference in the amount of inositol monophosphate that accumulated in the frontal cortex after the DOI treatment in the C57BL/6J mice. However, the pharmacokinetic characteristics of the DOI varied between the sexes; the female C57BL/6J mice had lower brain and plasma levels of DOI 30 and 60 min after treatment than the male C57BL/6J mice [102].

Understanding how sex affects cannabinoid pharmacology is crucial given the growing popularity of cannabis edibles for medicinal purposes and the continuous abuse of these products. Female rats appear to be more sensitive to several consequences of cannabinoid use, such as anti-nociception, discriminative stimulus, and reinforcing effects, according to the studies that have examined the sex differences in the behavioral effects of cannabinoids [103,104,105,106,107]. Interestingly, for the optimum acquisition and preservation of stimulus control in delta-9-tetrahydrocannabinol (THC) discrimination protocols, the female rats required a lower THC training dose than the male rats [106,108]. When the rats of either sex were trained to distinguish between identical THC doses, THC was also more effective at inducing discriminative stimulus effects in the female Sprague Dawley rats than in the males of the same strain. However, the THC potency to produce discriminative stimulus effects was comparable in the C57BL/6J mice of both the male and female sexes treated to distinguish 5.6 mg/kg of THC from the vehicle as well as in the mice trained to distinguish a higher dose of THC (30 mg/kg) [109]. In 2020, Wiley et al. investigated the effects of the intraperitoneal administration of THC and its main psychoactive metabolite, 11-OH-THC, in rodent models of psycho-activity and molecular assays of cannabinoid receptor type-1 pharmacology on the effects of age, sex, and rodent species. They observed that both 11-OH-THC and THC functioned as partial agonists in guanosine triphosphate, labeled on the gamma phosphate group with equal intensity in both the species and the male and female sexes [110].

Cannabinoids also demonstrated promise in the treatment of several challenging pain conditions and may provide a non-opioid alternative for the long-term control of chronic inflammatory pain [111,112]. In comparison to men, women report a higher prevalence of chronic pain as well as higher levels of experimentally produced and postoperative pain [113,114,115]. In a mouse model of inflammatory pain, LaFleur et al. observed that, in comparison to the male mice, the female mice had a lower susceptibility to the effects of Δ-9-THC and a synthetic cannabinoid. The attenuation of nociceptive behaviors for both agonists in both the male and female sexes was exacerbated by the S426A/S430A mutation. Compared to the male mice, the female mice showed a delayed tolerance to Δ-9-THC, whereas the S426A/S430A mutation caused a delayed tolerance in both the male and female sexes. Compared to the wild-type controls, the male S426A/S430A mutant mice exhibited a resistance to tolerance to the synthetic cannabinoid [116].

A preclinical investigation on female and male Sprague Dawley rats and male CD1 mice evaluated NKTR-181 (the novel mu-opioid receptor agonist NKTR-181 with a limited entry into the brain) preclinical pharmacology in comparison to commonly utilized mu-opioid receptor agonists; in the hot-water tail-flick test, NKTR-181 demonstrated dose- and time-related anti-nociception with peak effects comparable to morphine, without sex or species differences [117].

In preclinical diabetes research, animal models are crucial. As one of the most often carried out studies in metabolic research, glucose tolerance tests (GTTs) are used to test new antidiabetic therapies on raised blood glucose concentrations [118]. Female mice are frequently excluded from studies on diabetes because they lose some of their glucose intolerance and insulin resistance [119,120,121,122]. In fact, it is generally accepted that this relative lack of phenotypic behavior reduces their value in treatment efficacy trials by introducing preclinical bias and possibly obstructing translation to a different clinical population. It might also be the reason why researchers are hesitant to investigate female mice because of their perceived higher variability in blood glucose levels throughout the estrous cycle [123,124]. Despite a rising emphasis on the necessity to take into account sex as a biological variable in preclinical studies, the effect of sex and the estrous cycle on blood glucose fluctuation in the GTT has not previously been examined in depth [3,8,124]. The effects of sex, dosage method, length of fasting, and acute habituation stress was examined by Kennard and colleagues in relation to glucose tolerance test (GTT) measures used in the preclinical assessment of putative glucose-modulating therapies. When starting a fast, the researchers noticed that the female mice were less susceptible to human involvement. After a 6 h fast, both the male and female mice’s basal blood glucose levels stabilize more quickly when the bedding is kept intact while the cage base is changed. Sixteen hours of continuous fasting produced an inflated GTT response but a substantial basal hypoglycemia. Exendin-4 and metformin had a similar effect after GTT protocol optimization, with the female mice exhibiting a more moderate but repeatable GTT response [125].

For the treatment of obesity and diabetes, unimolecular peptides that target the glucagon-like peptide-1 (GLP-1) and glucose-dependent insulinotropic polypeptide (GIP) receptors (GLP-1/GIP co-agonist) are now being tested in clinical settings [126,127]. From rodent models of obesity to non-human primates and humans, their effectiveness to enhance body weight, glucose management, and lipid metabolism outperforms best-in-class GLP-1 monotherapies [126,127,128]. Sachs et al. sought to identify biomarkers to promote non-invasive metabolic monitoring of compound treatment efficacy and research of additional treatment effects on an individual basis despite sex-specific plasma proteome profiling differences. In comparison to mono-agonist therapies, the GLP-1R/GIPR co-agonist significantly reduced obesity, glucose intolerance, non-alcoholic fatty liver disease, and dyslipidemia in both the male and female mice. In comparison to mono-agonist treatments, proteome profiling differences in both the male and female mice indicated larger alterations in plasma proteins after the GLP-1/GIP co-agonist [129].

**Table 1:** Summary of the preclinical pharmacological studies which include sex evaluation.

Ref #	First Author	Year	Journal	Topic
[38]	Song, D.	2018	*Pharmaceutical Research*	Inflammation
[102]	Jaster, A.M.	2022	*Neuroscience Letters*	Psychiatry
[103]	Craft, R.M.	2013	*Life Sciences*	Cannabinoid
[104]	McGregor, I.S.	2007	*British Journal of Pharmacology*	Cannabinoid
[105]	Cooper, Z.D.	2018	*Neuropsychopharmacology*	Cannabinoid
[106]	Wiley, J.L.	2017	*Drug and Alcohol Dependence*	Cannabinoid
[107]	Craft, R.M.	2013	*Pain*	Cannabinoid
[108]	Winsauer, P.J.	2012	*Pharmacology, Biochemistry and Behavior*	Cannabinoid
[109]	Wiley, J.L.	2011	*Behavioural Pharmacology*	Cannabinoid
[110]	Wiley, J.L.	2021	*Progress in Neuro-Psychopharmacology & Biological Psychiatry*	Cannabinoid
[111]	Blake, D.R.	2006	*Rheumatology (Oxford)*	Cannabinoid
[112]	Johnson, J.R.	2013	*Journal of Pain and Symptom Management*	Cannabinoid
[113]	Nahin, R.L.	2012	*The Journal of Pain*	Pain
[114]	Riley, J.L.	1998	*Pain*	Pain
[115]	Aubrun, F.	2005	*Anesthesiology*	Analgesia
[116]	LaFleur, R.A.	2018	*Neuroreport*	Cannabinoid
[117]	Kopruszinski, C.M.	2021	*Cellular and Molecular Neurobiology*	Analgesia
[118]	Pacini, G.	2013	*Journal of Diabetes Research*	Endocrinology
[119]	Nyavor, Y.	2019	*Cell and Tissue Research*	Endocrinology
[120]	Kaikaew, K.	2019	*Endocrinology*	Endocrinology
[121]	Pettersson, U.S.	2012	*PLoS One*	Endocrinology
[122]	Rebolledo-Solleiro, D.	2018	*Physiology and Behavior*	Endocrinology
[123]	Bartke, A.	1973	*Endocrinology*	Endocrinology
[124]	Beery, A.K.	2018	*Current Opinion in Behavioral Sciences*	Endocrinology
[125]	Kennard, M.R.	2022	*Diabetes, Obesity and Metabolism*	Endocrinology
[129]	Sachs, S.	2021	*Diabetes, Obesity and Metabolism*	Endocrinology
[130]	Kremer, J.J.	2015	*Journal of Pharmacological and Toxicological Methods*	Cardiology
[131]	Bourdi, M.	2020	*Regulatory Toxicology and Pharmacology*	Cancer
[132]	Ewertz, M.	2015	*Acta Oncologica*	Cancer
[133]	Kotaka, M.	2020	*Cancer Chemotherapy and Pharmacology*	Cancer
[134]	Minami, T.	2020	*European Journal of Pharmacology*	Cancer
[135]	Earp, J.C.	2009	*Pharmaceutical Research*	Rheumatology
[136]	Dubois, D.C.	2008	*Journal of Pharmacology and Experimental Therapeutics*	Rheumatology
[137]	Earp, J.C.	2008	*Journal of Pharmacology and Experimental Therapeutics*	Rheumatology
[138]	Fletcher, C.V.	2014	*Proceedings of the National Academy of Sciences*	Antiretroviral drugs
[139]	Thompson, C.G.	2015	*Antimicrobial Agents and Chemotherapy*	Antiretroviral drugs
[140]	Cottrell, M.L.	2016	*The Journal of Infectious Diseases*	Antiretroviral drugs
[141]	Dimopoulos, Y.	2017	*Current HIV/AIDS Reports*	Antiretroviral drugs
[142]	Burgunder, E.	2019	*Journal of Pharmacology and Experimental Therapeutics*	Antiretroviral drugs

# reference number.

In a study on dog models by Kremer et al. beagles that were given etilefrine, sotalol, and hydralazine were implanted with a miniature telemetry blood pressure transmitter to monitor their blood pressure. Both the males and female beagles reported changes in blood pressure as a consequence of the etilefrine. Both the hydralazine and sotalol had similar effects on both the male and female beagles and continued for 19 h post-dose. The exposure levels were dose-dependent and quantifiable between 7 and 7.5 h post-dose in the dogs that were given etilefrine, sotalol, and hydralazine. The male and female beagles exposed to 10 mg/kg of etilefrine experienced different concentrations (169 vs. 268 ng/mL or 69%), but generally, there were only small differences [130].

Bourdi and colleagues conducted in vitro and in vivo studies on metarrestin safety evaluation in beagles; they observed a dose-related increase in systemic exposure with no sex difference on days 1 and 27. From day 1 to day 27, metarrestin accumulated in both the male and female dogs and at all dose levels. No adverse effects were reported on the other days’ dosage for 28 days; the level in the dogs was estimated to be 0.25 mg/kg of metarrestin, with a mean male and female maximum concentration of 82.5 ng/mL and an exposure of 2521 h·ng/mL on day 27 [131].

The use of the platinum-based chemotherapeutic oxaliplatin for the treatment of colorectal cancer comes with a major dose-limiting adverse effect, peripheral neuropathy [132]. In a recent clinical phase 2 research, thrombomodulin alfa, a recombinant human soluble thrombomodulin, was demonstrated to inhibit oxaliplatin-induced peripheral neuropathy [133]. A preclinical pharmacology on rats intravenously treated with oxaliplatin (6 mg/kg) was conducted; treatment-induced mechanical hyperalgesia was inhibited by a single intravenous infusion of thrombomodulin alfa in a dose-dependent manner, with no sex-related differences in the effectiveness [134].

Preclinical pharmacologic studies on rheumatoid arthritis frequently employ collagen-induced arthritic rats [135,136,137]. In the first analysis on sex differences in collagen-induced arthritic rats, the male rats reported 43% larger dexamethasone clearances; in the female rats, the temporal patterns of paw edema showed earlier development, earlier times of peak edema, and earlier illness remission. In both the male and female rats with comparable capacity values, dexamethasone effectively reduced paw edema, but the dexamethasone potency was lower in the females [38].

Low-level viral replication within tissues as a result of insufficient antiretroviral (ARV) penetration is one potential reason of viral rebound [138]. Studies on therapeutic agent concentrations in colorectal and female genital tract tissues revealed significantly varied ARV penetration in the field of HIV prophylaxis [139,140]. Even though these tissues are essential to the pathogenesis of HIV, there are a lot fewer data on medication exposure for the presumed lymph node reservoir [141]. Preclinical models including HIV-infected humanized mice and nonhuman primates (NHPs) with reverse transcriptase simian/HIV revealed that sex had no impact on the ARV pharmacokinetics in the collected lymph [142].

## 7. Why There Is the Need to Include Female Models

In general, a survey of the literature showed that preclinical research has mainly employed males (80%). The 44% of the papers on preclinical research models for medications created for diseases mostly affecting women did not mention the sex of the animals utilized; of those that did, 88% researched solely male animals [58,60]. The poor translation of animal findings to humans was therefore attributed to sex bias in preclinical investigations [7]. Although regulatory toxicity studies must use both the male and female sexes (perhaps as compensation?) prior to the first in-human studies, the scientist is required to “consider” sex when designing safety pharmacology studies that look at the short-term side effects on physiological functions. However, animal models have other drawbacks besides sex bias.

There are other factors than the X and Y chromosomes that distinguish females from males. There has been a prevalent, false notion that male and female rodents have similar characteristics in preclinical investigations [124]. Males and females differ in a variety of physiological phenotypes, including basic physiological traits such as body weight, lean and fat mass, as well as several neuroendocrine, immunological, and behavioral traits outside of reproductive behaviors [84,97]. Additionally, many human diseases have different effects on men and women, and sex differences can have an impact on treatment effectiveness, symptom progression, and disease susceptibility. Cardiovascular illness, autoimmune disorders, chronic pain, and neuropsychiatric disorders have well-established sex disparities, with females typically experiencing higher incidences than males [65]. A study design, phenotypes, pharmacokinetics, pharmacodynamic measurements, or the interpretation of results without considering sex as a covariate are all examples of areas where accuracy in reporting the data is lacking [8,9,10,15,16,20,23,26,27,37,41,48,74,86]. Sex distinctions in disease onset and development have also been noted in relation to animal models used in pharmacological investigations. Furthermore, it should be useful to consider sex distinctions in disaggregated data by age. For humans, especially for women, different periods of life can influence health outcome and treatment response. A preclinical research model that is truly transferable to human reality should also consider the age variable [143,144,145].

## 8. What Risks May Arise from Continuing Female Exclusion from Experimental Design

There have been ups and downs in the sex/gender debate throughout history, such as when the FDA banned women who may become pregnant from participating in phase 1 and phase 2 trials in response to the thalidomide (Contergan) and diethylstilbestrol scares in 1977. After studies revealed that women were underrepresented in clinical studies, revisions to these recommendations were made in 1993. From 1997 to 2000, eight out of ten prescription drugs were then taken off the market because they posed a greater health risk to women [146,147].

Sex is a crucial biological factor that has significant effects. Preclinical research on females has underrepresented female cells and animals, which led to a worse understanding of the pathophysiological, physiological, and biochemical pathways in females than in males. It is impossible to know whether the results acquired in male cells and animals also apply to female cells and animals without data from females [5,7,148,149]. The pipeline for developing new therapies and finding new molecules is provided by basic science. The application of these preclinical discoveries to the health of men and women is therefore impacted by the discovery of sex differences in pathophysiologic pathways in animal models of disease [150].

The interpretation of sex differences in scientific results presents difficulties, and the field is burdened by inconsistent results. The results could be affected by factors such as rat age and strain; in addition, the data could be susceptible to things such as the time of day that the data were collected and methodological variations between laboratories [149,151,152]. Estrus cycle determination and its appraisal in result interpretation are crucial for furthering the investigation of sex differences with the involvement of a male comparison group. Additionally, the continued use of studies on hormonal and neutering treatments adds to the expanding understanding and appreciation of the significance of sex differences in pharmacology. However, the exclusion of female animals from preclinical studies may lead to the waste of resources on treatments that inevitably do not receive approval. Increased sample sizes will be needed in studies that include females, but the effect of sex on pathological processes and treatment responses shows that differences between the sexes are crucial factors to consider in preclinical research.

## 9. What about Transgender Models?

If preclinical research still hesitates to enroll the female model, it is almost absent in terms of the study of diseases and drug response in the context of the transgender model. Yet, data from the international scientific literature suggest that the percentage of the transgender population is between 0.5 and 1.2% of the total population. In Italy, for example, the transgender population consists of 400,000 people [153]. There must therefore be a growing interest of the scientific world regarding the health of transgender people. Especially in recent years, several works have been published on this segment of the population, but the small number of subjects studied does not allow us to reach certain conclusions regarding the susceptibility and risk factors for chronic–degenerative diseases not allowing a specific health plan in these population groups. Preclinical research must also adapt to this as soon as possible, starting to propose an experimental transgender model in which to test when a mixed pool of hormones can influence the course of diseases and responses to therapy. The scientific literature related to preclinical research in this field is currently scarce.

While transgender individuals frequently receive gender-affirming/confirming hormone therapy, no hormonal agents or clinical protocols for transgender medicine have yet received approval from international medical regulatory agencies, such as the European Medicines Agency or the Food and Drug Administration. “Off-label” hormone therapies are thus based on recommendations from Endocrine Societies or organizations of a similar nature [154]. The primary goals of human hormone therapies, which are often given throughout life, are to reduce secondary sex traits and return sex hormone levels to the normal range for the matching cisgender. For transgender men, hormone therapy entails injecting testosterone (intramuscularly or subcutaneously); more recently, transdermal administration (patches or gel) of a longer acting form has been suggested because it is appropriate for long-term use. For transgender women, hormone therapy typically consists of β-estradiol that is given transdermally, orally, or intravenously, either on its own or in combination with medications that suppress androgen levels. Depending on how β-estradiol is administered, different dosage levels and administration windows apply. Cyproterone acetate (50 mg daily) is the most widely used anti-androgen medication in Europe [155]. There is a lack of information currently available regarding the potential impacts of HT on the health of transgender individuals as well as its potential long-term consequences. Since one of the main objectives of risk assessment is to characterize the chemical risks in potentially sensitive sub-population groups and to ensure the selection of appropriate safety factors [155], toxicological issues should also be included in this context. Endocrine disruptors, which share targets and modes of action with hormone therapy, are among the environmental and food pollutants that transgender people, like the general population, are exposed to on a regular basis, making transgender people a subgroup of the population that is more susceptible and vulnerable to their effects. To accurately perform a danger identification for transgender people undergoing hormone therapy in this context, specialized animal models should be established and employed [155].

Targeted animal models must be used in toxicological investigations in order to gather reliable information for identifying chemical hazards [156]. People with TG who underwent HT demonstrated distinctive characteristics in terms of particular susceptibility and vulnerability to chemical contaminants; as a result, they require appropriate animal models based on pertinent and novel biomarkers [157].

A significant model for determining whether the sex variations in the phenotypes are brought on by the complement of the sex chromosomes (XX vs. XY), gonadal hormones, or both, is the “four core genotypes” (FCG) mouse model. In the model, a Sry transgene is inserted onto an autosome after the testis-determining gene Sry is deleted from the Y chromosome. In order to compare XX and XY mice with the same type of gonad and determine the phenotypic consequences of sex chromosomal complement in cells and tissues, it breeds XX and XY mice with testes and XX and XY mice with ovaries [158,159,160,161,162,163]. The number of X chromosomes (including X dose, X imprint, or indirect effects of X inactivation), the presence or absence of the Y chromosome, or both, may be the cause of a sex chromosome effect (XX not equal to XY) in FCG mice [159,164]. So, to distinguish between these options, the XY* model is helpful. In XY* mice, which were first discovered by Eicher et al. a defective pseudo-autosomal region on the Y chromosome recombines improperly with the X chromosome [160,162]. When XX females and XY* fathers are mated, mice comparable to XX and XO gonadal females, as well as XY and XXY gonadal males, are produced [159]. When comparing XO vs. XX females or XY vs. XXY males, one can determine the impact of having one X chromosome against two. Comparisons of XY vs. XO and XXY vs. XX are used to assess the impact of having one Y chromosome vs. none. Mice with a Y chromosome are gonadal males according to the XY* paradigm.

A very recent study evaluated the ovarian dynamics after testosterone cessation using a mouse model that mimicked trans-masculine testosterone therapy. During six weeks, they administered testosterone enanthate or a vehicle control injection of 0.9 mg once per week to post-pubertal female C57BL/6N mice 9–10 weeks of age. Within 1 week of beginning the testosterone treatment, all the testosterone-treated mice ceased cycling and displayed chronic diestrum, whereas the control mice cycled regularly. The age-matched vehicle-treated diestrum controls and a group of mice treated with testosterone for 6 weeks each were slaughtered. The age-matched vehicle-treated controls and a different group of mice that had been receiving testosterone therapy were sacrificed together in diestrum four cycles after the cycle had resumed. Comparing the post-testosterone group to both the age-matched controls and the mice at 6 weeks on testosterone demonstrated stromal alterations with clusters of big round cells. Periodic acid-Schiff staining, which was documented in multinucleated macrophages in aging mouse ovaries, was present in these clusters. A significant number of these cells also showed staining for the macrophage markers CD68 and CD11b. Comparing the age-matched controls and ovaries at 6 weeks on testosterone, the ovarian ribonucleic acid-sequencing revealed that the immunological pathways were upregulated post-testosterone [165].

## 10. Take Home Message

The inclusion of both the male and female sexes is a valid approach to improve heterogeneity [149]. If researchers fail to disclose the sex utilized, or if they let sex be an uncontrolled variable throughout the experiment, or if they omit to account for sex during the study and sex is a substantial source of variance, sex bias may contribute to difficulties in replication.

Female cells function differently from male cells, which affects how sensitive they are to stressors and how susceptible they are to disease. This has an impact on how they respond to medication. Therefore, research on gender disparities and cell injury mechanisms seems essential for creating novel, more effective therapeutic approaches.

The capacity of scientists to properly consider these variations in diseases and patient populations is essential to the success of drug development. This subsequently enables us to create new pharmaceuticals by starting a symphony of preclinical research and safety studies customized precisely to the features of the new drug, and to wisely recognize where sex-specific considerations are required.

## 11. Conclusions

The knowledge of sex influence and differences in pharmacological studies should be taken into account in terms of treatment personalization and precision medicine.

Despite the fact that many diseases are known to display sexual dimorphism, the majority of preclinical research studies do not consider sex. Sex-based statistical analyses are still rare in preclinical investigations. In addition, despite the well-known sexual dimorphism, women are frequently underrepresented in various therapeutic trials. A delay in the diagnosis and treatment for both the male and female sexes may also result from societal representations of certain pathologies as “male” or “female” diseases, which are sensitive to social representations of certain pathologies. Finally, there may be sex differences in the efficacy and safety of numerous pharmacological classes. For instance, as a result of differing pharmacokinetic characteristics, women present more adverse effects than men. To improve the translation of observed results and advance customized therapy, sex must be taken into account as a variable starting in the preclinical stage [166].

Further works which aim to highlight sex differences in specific areas/diseases are needed to deeply explore different therapeutic strategies.

## Data Availability

Data sharing not applicable.
